# Supplementary figures and images for: Dynamic centriolar localization of Polo and Centrobin in early mitosis primes centrosome asymmetry
Source: PLoS Biol. 2020 Aug 6;18(8):e3000762. doi: 10.1371/journal.pbio.3000762 (PMC7433902; doi:10.1371/journal.pbio.3000762)

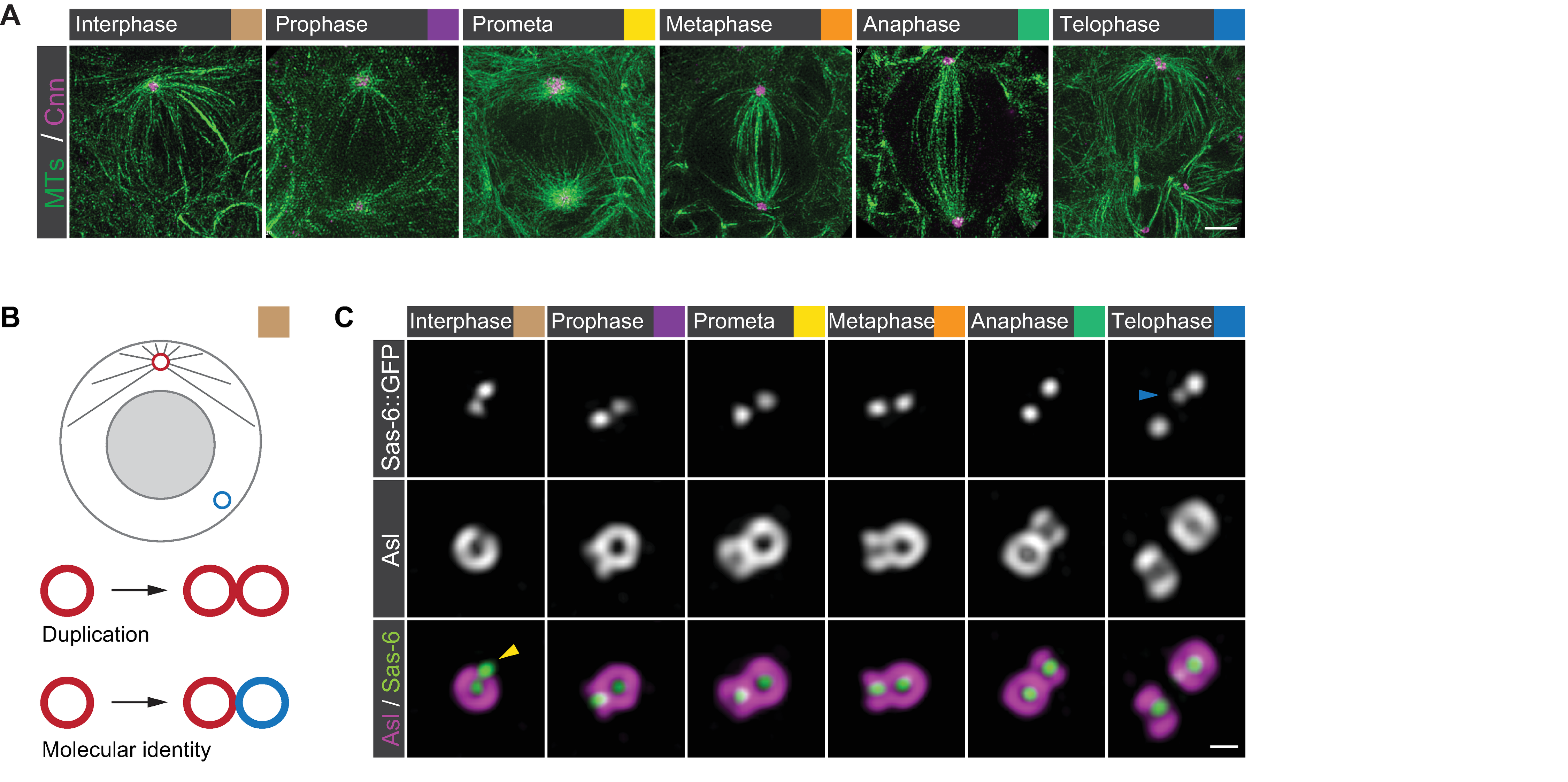

Supplement: S1 Fig — (A) Representative 3D-SIM images of neuroblasts expressing the pericentriolar marker Cnn::GFP stained for α-Tubulin, labelling MTs (green). The morphology of the microtubule array and cell shape were used to define neuroblast cell-cycle stages. (B) Neuroblast centrosomes are inherently asymmetric in interphase but when neuroblast centrioles duplicate and acquire a unique molecular identity (indicated by arrow and color switch) is unknown. (C) Representative interpolated 3D-SIM images of third instar larval neuroblast centrosomes, expressing Sas-6::GFP (top row; white. Green in merge) and stained for Asl (middle row; white. Merged channels; magenta). The yellow arrowhead highlights the cartwheel of the forming centriole. Cartwheel duplication can be observed at the telophase to interphase transition, concomitantly with centrosome separation (blue arrowhead). Cell-cycle stages are indicated with colored boxes. Scale bar is 3 μm in panel A and 0.3 μm in panel C. GFP,; MT, microtubule; 3D-SIM, 3D structured illuminated microscopy. (TIF) [file pbio.3000762.s001.tif]

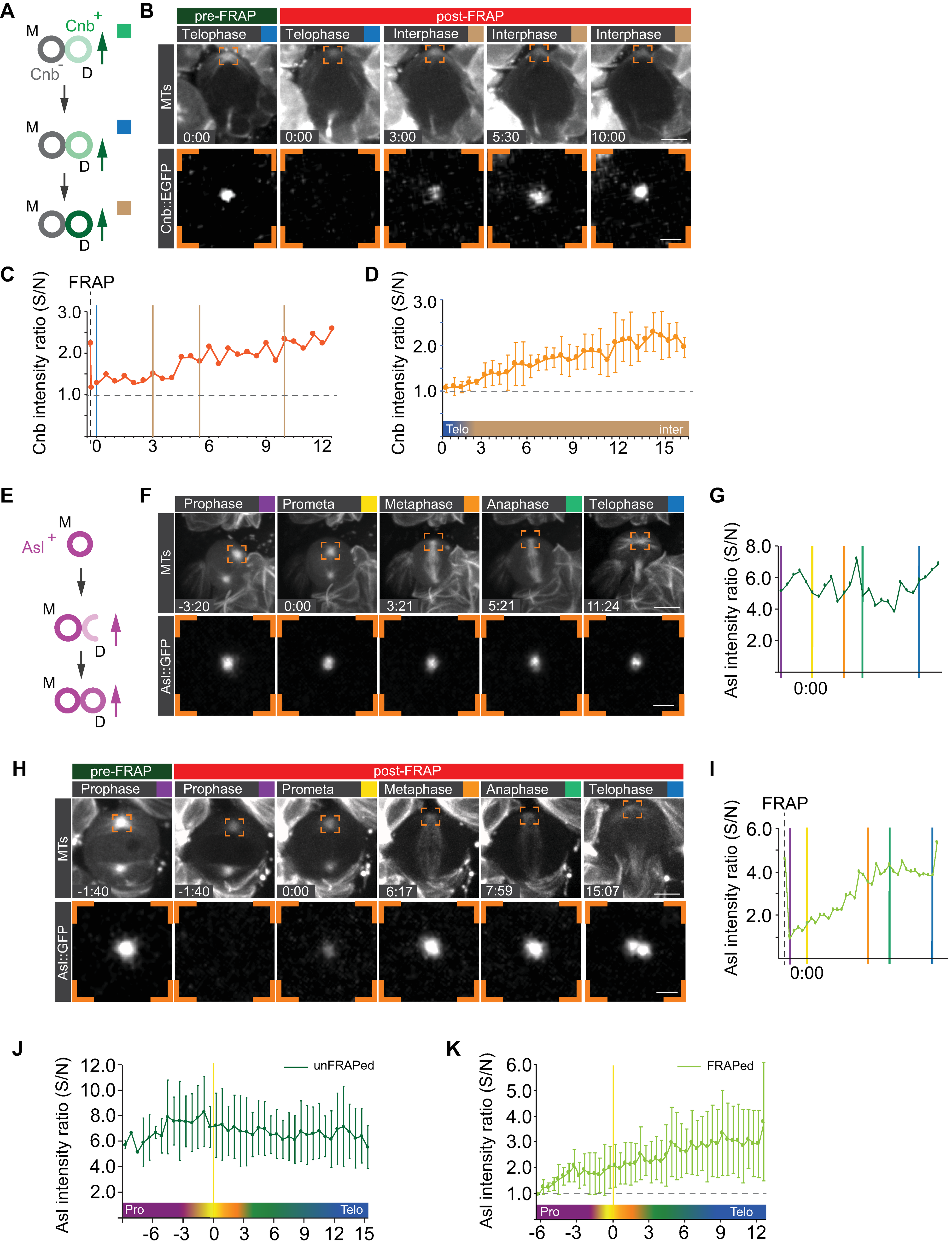

Supplement: S2 Fig — (A) Cnb enrichment at the end of mitosis could occur through new recruitment of cytoplasmic Cnb (vertical green arrows). (B) Representative image sequence and (C) intensity profile of a FRAPed wild-type neuroblast expressing endogenously tagged Cnb::EGFP (white; bottom row) together with the MT marker mCherry::Jupiter (white; top row). The orange brackets highlight the apical centrosome where Cnb::EGFP (bottom row) is measured. FRAPing occurred in late telophase. Colored vertical bars in the intensity plot indicate specific cell-cycle stages. The vertical dashed line refers to the time point when bleaching was performed. (D) Mean intensity plot of 8 FRAPed apical centrosomes in Cnb::EGFP expressing neuroblasts. Error bars indicate standard deviation of the mean. Cnb intensity was plotted as a ratio of centrosomal Cnb/cytoplasmic Cnb (S/N; see Methods) in panels C and D. (E) Asl enrichment throughout mitosis could occur through constant loading of cytoplasmic Asl (magenta vertical arrows). Representative unFRAPed (F) and FRAPed (H) wild-type neuroblast expressing Asl::GFP (white; bottom row) together with the MT marker mCherry::Jupiter (white; top row). The orange brackets highlight the apical centrosome where Asl::GFP (bottom row) is measured. Intensity profile of the unFRAPed (G) and FRAPped (I) apical Asl::EGFP signal of the neuroblasts shown in panels F and H. Colored vertical bars indicate specific cell-cycle stages. The vertical dashed line refers to the time point when bleaching was performed. Mean intensity plot of 9 unFRAPed (J) and 8 FRAPed (K) apical centrosomes for neuroblasts expressing Asl::GFP. For panels G, I, J, and K, Asl intensity was plotted as a ratio of centrosomal Asl/cytoplasmic Asl (S/N; see Methods). Error bars indicate standard deviation of the mean. Time scale is mm:ss. Scale bar in panels B, F, and H is 5 μm (top row) and 1 μm (bottom row). The data presented here were obtained from 3 independent experiments. Numerical data for panels C, [file pbio.3000762.s002.tif]

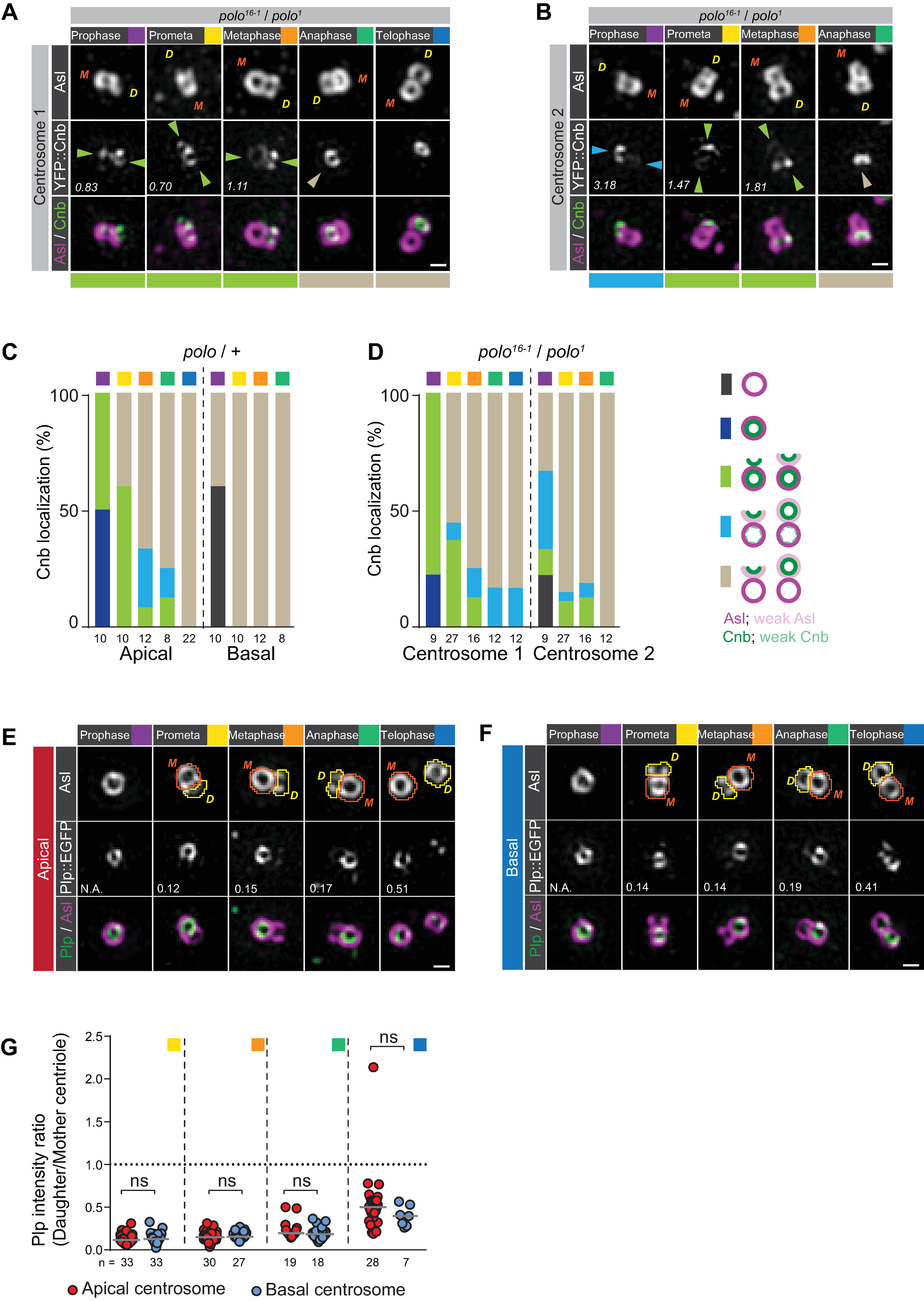

Supplement: S3 Fig — Representative 3D-SIM images of the (A) first and (B) second centrosome of a polo mutant (polo1/polo16-1) third instar larval neuroblast expressing YFP::Cnb (white; middle row, green; bottom row) and stained for Asl (white; top row, magenta; bottom row). Yellow “D” and orange “M” refer to daughter and mother centrioles based on Asl intensity. polo mutant neuroblasts show a loss of MTOC activity during interphase, which randomizes centrosome positioning and distribution. Because we cannot distinguish between the “apical” and “basal” centrosomes anymore we refer to centrosome 1 and 2 instead; centrosome 1 being the most affected regarding Cnb mislocalization. Colored arrowheads and bars underneath the images highlight the degree of Cnb centriolar asymmetry. Graphs showing the timeline of Cnb’s centriolar asymmetry at defined mitotic stages in (C) control (polo/+) and (D) polo mutant (polo1/polo16-1) neuroblasts. The bars show the percentage of neuroblasts containing a single Cnb+ centriole (dark blue), a single centriole without Cnb (dark gray), Cnb on both centrioles (transition stage with a daughter/mother ratio < 2; light green), predominant Cnb localization on the daughter centriole (strong asymmetry with a daughter/mother ratio between 2 and 10; light blue) or in which Cnb is completely shifted to the daughter centriole (complete asymmetry with a daughter/mother ratio > 10; light brown). This experiment was done 3 times. Representative 3D-SIM images of (E) apical and (F) basal third instar larval neuroblast centrosomes, expressing Plp::EGFP (white in middle row, green in merge), co-stained with Asl (white on top, magenta in merge). Orange and yellow shapes represent mother “M” and daughter “D” centrioles, respectively, based on Asl intensity. The number represents total Plp intensity ratios (daughter/mother centriole) in the shown image. Plp asymmetry ratios for the apical (red dots) and the basal (blue dots) centrosome are plotted in panel G from 3 independent e [file pbio.3000762.s003.tif]

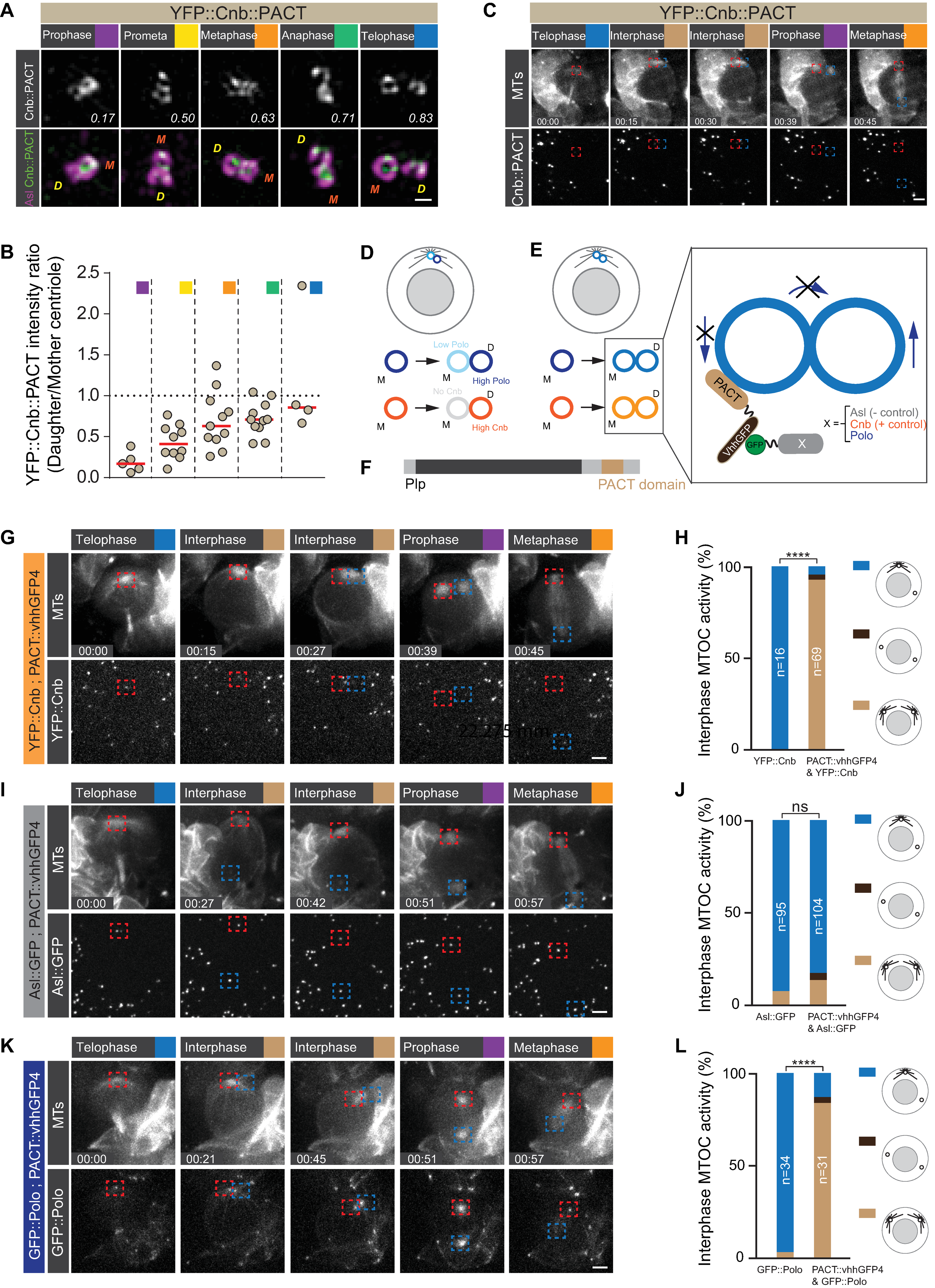

Supplement: S4 Fig — (A) Representative 3D-SIM images of third instar larval neuroblast centrosomes, expressing YFP::Cnb::PACT (white in the first row, green in the merge) and stained for Asl (magenta in the merge). The number represents total YFP::Cnb::PACT intensity ratios (daughter/mother centriole) in the shown image. YFP::Cnb::PACT intensity ratios (daughter/mother centriole) are plotted in panel B. Yellow “D” and orange “M” refer to daughter and mother centrioles based on Asl intensity. (C) Representative live-cell imaging series from a neuroblast, recorded in the intact brain, expressing the microtubule marker mCherry::Jupiter (MTs, first row) and YFP::Cnb::PACT (second row). 3D-SIM and live-imaging experiments were performed 2 times each. (D) To test the function of centriolar asymmetry establishment, the asymmetric centriolar localization of Polo and Cnb needs to be perturbed. (E) The vhhGFP4 nanobody specifically traps GFP or YFP tagged proteins and was tethered to the mother centriole using Plp’s PACT domain (F). Upon co-expression with GFP or YFP tagged centrosomal proteins in neuroblasts, centriolar asymmetry establishment was impaired (crossed-out arrows shown for Polo; blue). Representative live-cell image series from intact brains for neuroblasts expressing the microtubule marker mCherry::Jupiter (first row) and PACT::VhhGFP4 together with (G) YFP::Cnb, (I) Asl::GFP, and (K) GFP::Polo trans-gene (genomic rescue construct; see Methods). MTOC phenotype quantifications are shown for (H) YFP::Cnb, (J) Asl::GFP, and (L) GFP::Polo (blue; wild-type–like asymmetry, dark brown; loss of MTOC activity, light brown; gain of MTOC activity). Fisher’s exact test: YFP::Cnb with and without PACT::VhhGFP4 expression; p = 1.874 × 10^−14^. Asl::GFP with and without PACT::VhhGFP4 expression; p = 0.1751. GFP::Polo with and without PACT::VhhGFP4 expression; p = 1.105 × 10^−12^. Cell-cycle stages are indicated with colored boxes. The data presented here were obtained from 2, 4, and 3 independent ex [file pbio.3000762.s004.tif]

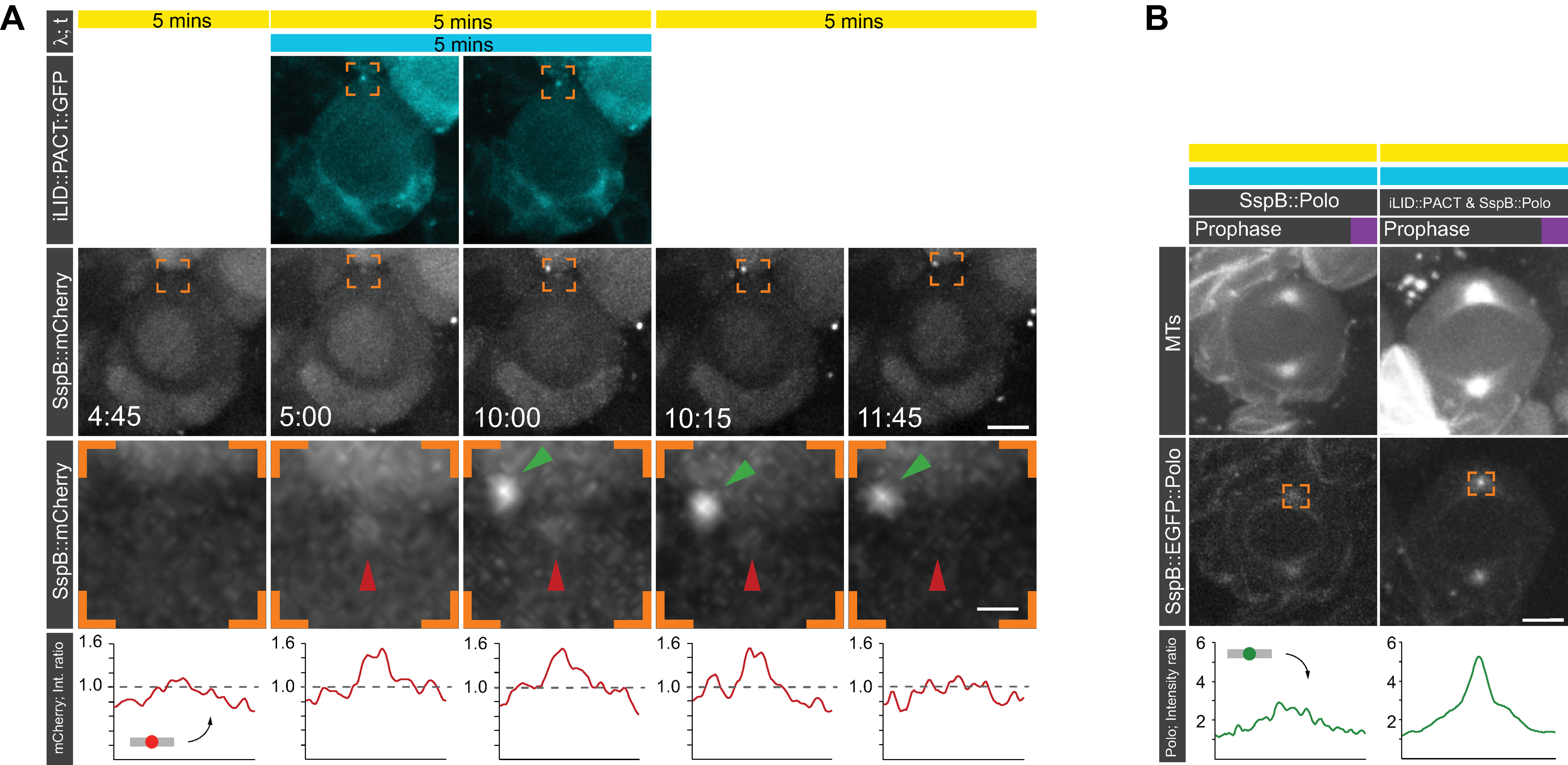

Supplement: S5 Fig — (A) Representative time-lapse frames of a third instar neuroblast—imaged in an intact brain—expressing SspB::mCherry (second and third row; gray) together with iLID::PACT::GFP (cyan; top row). Light exposure regime is indicated on the top. Orange brackets and red arrowheads highlight the apical neuroblast centrosome. An unrelated mCherry particle is highlighted with the green arrowhead. Intensity ratios, displaying the ratio of centrosomal/cytoplasmic SspB::mCherry are shown below; SspB::mCherry intensity was measured along a 12-pixel wide line covering the centriole and normalized against cytoplasmic mCherry levels. Note that SspB::mCherry is recruited from the cytoplasm to the apical centrosome within 5 seconds and released back into the cytoplasm within approximately 2 minutes. (B) Representative Prophase time-lapse frames of third instar larval neuroblasts expressing SspB::EGFP::Polo alone (control; left) or in conjunction with iLID::PACT::HA (right). SspB::EGFP::Polo (middle row; white) appears enriched and more focused in the presence of iLID::PACT::HA and after blue-light exposure. Intensity ratios, displaying the ratio of centrosomal/cytoplasmic SspB::EGFP::Polo are shown below; SspB::EGFP::Polo intensity was measured along a 12-pixel wide line covering the centriole and normalized against cytoplasmic EGFP levels. Time scale is mm:ss. Scale bar is 1 μm for the third raw of panel A and 5 μm for other images. EGFP, Enhanced green fluorescent protein; GFP, green fluorescent protein; iLID, improved light-induced dimer; PACT, Pericentrin-AKAP-450 containing targeting; SspB, Stringent starvation protein B. (TIF) [file pbio.3000762.s005.tif]
