# Supplementary material for: Dynamic centriolar localization of Polo and Centrobin in early mitosis primes centrosome asymmetry
Source: PLoS Biol. 2020 Aug 6;18(8):e3000762. doi: 10.1371/journal.pbio.3000762 (PMC7433902; doi:10.1371/journal.pbio.3000762)
Supplement: Attachment — Submitted filename: Reviewers comments_May29_FINAL.docx [file pbio.3000762.s015.docx]

*Copyediting*
Upon acceptance of your article, your final files will be copyedited and typeset into the final PDF. While you will have an opportunity to review these files as proofs, PLOS will only permit corrections to spelling or significant scientific errors. Therefore, please take this final revision time to assess and make any remaining major changes to your manuscript.

NOTE: If Supporting Information files are included with your article, note that these are not copyedited and will be published as they are submitted. Please ensure that these files are legible and of high quality (at least 300 dpi) in an easily accessible file format. For this reason, please be aware that any references listed in an SI file will not be indexed. For more information, see our Supporting Information guidelines:
<https://journals.plos.org/plosbiology/s/supporting-information>

*Published Peer Review History*
Please note that you may have the opportunity to make the peer review history publicly available. The record will include editor decision letters (with reviews) and your responses to reviewer comments. If eligible, we will contact you to opt in or out. Please see here for more details:
<https://blogs.plos.org/plos/2019/05/plos-journals-now-open-for-published-peer-review/>

*Early Version*
Please note that an uncorrected proof of your manuscript will be published online ahead of the final version, unless you opted out when submitting your manuscript. If, for any reason, you do not want an earlier version of your manuscript published online, uncheck the box. Should you, your institution&apos;s press office or the journal office choose to press release your paper, you will automatically be opted out of early publication. We ask that you notify us as soon as possible if you or your institution is planning to press release the article.

*Protocols deposition*
To enhance the reproducibility of your results, we recommend that if applicable you deposit your laboratory protocols in protocols.io, where a protocol can be assigned its own identifier (DOI) such that it can be cited independently in the future. For instructions see: <https://journals.plos.org/plosbiology/s/submission-guidelines#loc-materials-and-methods>

*Submitting Your Revision*
To submit your revision, please go to <https://www.editorialmanager.com/pbiology/> and log in as an Author. Click the link labelled &apos;Submissions Needing Revision&apos; to find your submission record. Your revised submission must include a cover letter, a Response to Reviewers file that provides a detailed response to the reviewers&apos; comments (if applicable), and a track-changes file indicating any changes that you have made to the manuscript.

DATA POLICY: IMPORTANT - PLEASE READ

You may be aware of the PLOS Data Policy, which requires that all data be made available without restriction: <http://journals.plos.org/plosbiology/s/data-availability>. For more information, please also see this editorial: <http://dx.doi.org/10.1371/journal.pbio.1001797>

Note that we do not require all raw data. Rather, we ask that all individual quantitative observations that underlie the data summarized in the figures and results of your paper be made available in one of the following forms:

1) Supplementary files (e.g., excel). Please ensure that all data files are uploaded as &apos;Supporting Information&apos; and are invariably referred to (in the manuscript, figure legends, and the Description field when uploading your files) using the following format verbatim: S1 Data, S2 Data, etc. Multiple panels of a single or even several figures can be included as multiple sheets in one excel file that is saved using exactly the following convention: S1_Data.xlsx (using an underscore).

We have included the source data as an excel file in the revised submission. All source data is referenced in the main text and figure legends.

2) Deposition in a publicly available repository. Please also provide the accession code or a reviewer link so that we may view your data before publication.

Regardless of the method selected, please ensure that you provide the individual numerical values that underlie the summary data displayed in the following figure panels as they are essential for readers to assess your analysis and to reproduce it:

Fig. 1E, F, G; Fig. 2C, E, F; Fig. 3B, G; Fig. 4C; Fig. 5C, E; Fig. 6B, E, F; Fig. 7D, E; Fig. S2C, D, G, I, J, K; Fig. S3C, D, G and Fig. S4B, H, J, L

NOTE: the numerical data provided should include all replicates AND the way in which the plotted mean and errors were derived (it should not present only the mean/average values).

Please also ensure that figure legends in your manuscript include information on WHERE THE UNDERLYING DATA CAN BE FOUND, and ensure your supplemental data file/s has a legend.

Please ensure that your Data Statement in the submission system accurately describes where your data can be found.

Reviewers’ comments

Rev. 1:
This study by Gallaud and colleagues investigates the mechanisms regulating centrosome asymmetry and biased centrosome segregation in Drosophila neural stem cells. Using a number of elegant techniques including genetic models, superresolution and live imaging, nanobody and optogenetic trapping, they demonstrate that the mitotic kinase Polo and its centriolar substrate Centrobin accumulate on the daughter centriole during mitosis, generating molecularly distinct mother and daughter centrioles. They discover that Centrobin’s asymmetric localization may involve a direct re-localization mechanism, from mother to newly forming daughter centrioles, and is regulated by Polo-mediated phosphorylation. By manipulating the localization of these proteins, they propose that the establishment of centriole asymmetry in mitosis confers biased interphase centrosome activity, which is critical for correct spindle orientation.

Overall, the story is very intriguing, consistent with prior data, and the conclusions support the results. The work is very well performed, using multiple approaches to tackle each question. The data are presented clearly and are convincing. As such, I believe it is absolutely suitable for publication in PLoS Biology. I only have a few minor questions and concerns, which can be addressed by modifying the manuscript:

* Fig2 and page 10 - Based on the photobleaching experiments, the authors say that "In conclusion, we think that our data are most consistent with a hybrid model and propose that a small fraction of Cnb transfers from the mother to the daughter centriole in early mitosis through an unknown mechanism. From anaphase onward, the daughter centriole recruits additional Cnb from sources other than the mother centriole (Fig.2g)". Although this is a reasonable conclusion, an alternative interpretation would be that the reduction in Cnb levels on the mother centriole has nothing to do with its translocation to the daughter per se. Since the daughter centriole is templated off the wall of the mother centriole, one possibility is that the amount of Cnb in that (proximal) half of the mother centriole could be "displaced" as the cartwheel of the new procentriole forms. Furthermore, can the authors speculate as to why cells would need to go through this translocation? I&apos;m assuming there
is plenty of Cnb expressed and available to the forming procentriole… what would be the advantage of this translocation?!

The ’displacement’ model is attractive and could be a mechanism for the localization dynamics in early mitosis. However, according to this model, Cnb levels should remain constant throughout mitosis but our live cell imaging data reveal a drop in Cnb intensity from Prophase until Anaphase. Thus, some Cnb must be displaced from the centrosome during that time window. To get more insight into Cnb’s localization dynamics, we tagged Cnb endogenously with Dendra for photoconversion experiments with the goal to photoconvert the apical centrosome in early prophase and then monitor whether the fraction of converted Cnb (originally associated to the mother centriole) will be retained by the daughter centriole. However, the fluorescence of Dendra could not be detected, even before photoconversion.

We also think that Cnb is not strongly expressed for the following reasons: (1) Cnb::EGFP generated by CRISPR/Cas9 is quite dim and Dendra::Cnb could not be detected (Dendra is much dimmer than EGFP). (2) YFP::Cnb expressed under a polyubiquitin promoter (moderate expression), tends to form aggregates, a sign that a part of YFP::Cnb is actually not incorporated in the centriole.

* Fig 5B - what was the rationale for using a Cnb RNAi approach instead of the Cnb-phospho-mutant or hypomorphic strains?

We aimed for a clean depletion of Cnb protein as opposed to using a hypomorph or phospho-mutant. The Cnb RNAi line perfectly mimics the mutant phenotype (Januschke et al., NCB 2013). Using the RNAi line makes the experiment more convenient from a genetics standpoint.

* Fig 5 - The Cnb-PACT experiments are intriguing and show reversal of centriole asymmetries, at least with regards to Polo. Did the authors look at other centrosome maturation factors/PCM proteins to see if those are also reversed? I bring this up because in mammals, there appears to be an inverse relationship between asymmetrically localized, daughter centriole-enriched proteins (e.g. Cep120 or Neurl4) and the amount of PCM at that specific centriole (see for example Betleja et al, eLife 2018 or Li et al, EMBO Reports 2012). Thus, a mutual-exclusion model has been proposed. The authors may want to discuss those finding in the context of these data.

We have not investigated other PCM maturation factors. We analyzed the localization of Plp and found it to be inversely localized to Polo and Cnb (Figure 4D,E and Figure S3E-G). Plp has been described to extend from the centriolar wall into the pericentriolar matrix (Mannella et al., NCB 2012), We have included a sentence in the manuscript comparing it to the inverse relationship between Cep164 and Cep120 or Neurl4 and Cep170.

* Fig. 6 and 7 - I was a little bit confused about the outcomes of the experiments using nanobody trapping and optogenetic trapping. In the first case (Fig 6) the authors indicate that "preventing the normal establishment of Cnb and Polo asymmetry using the PACT domain perturbs biased MTOC activity in interphase", while in the latter (Fig 7) they suggest "perturbing normal Cnb and Polo asymmetry during mitosis disrupts asymmetric MTOC behavior in the following interphase". What I was waiting for was the phenotypic consequences of such manipulations! Was there a defect in segregation of cell fate markers? Did these manipulations disrupt asymmetric cell division? Were there any developmental defects associated with these, and are those consistent with what has previously been shown upon disruption of such processes?

These are important and interesting questions, which are beyond the scope of this manuscript. As far as we can tell, asymmetric cell division is not perturbed after nanobody or optogenetic manipulation, suggesting that cell fate determinants are also correctly segregated. These findings are in line with the published literature, using Drosophila neuroblast as a model to study centrosome asymmetry. Strong spindle misalignment can result in failed asymmetric cell division (e.g Cabernard & Doe, Dev Cell, 2009), but the manipulations shown here seem to induce a correction mechanism during mitosis causing the mitotic spindle to realign. We do not know the molecular nature of this mechanism.

* In the discussion, the authors do a very nice job of summarizing their results and putting them in context with previous work done in Drosophila. However, what is lacking is how these findings compare to the extensive data from mammalian cells with regards to asymmetrically localized proteins. I would highly encourage the authors to modify the Discussion section, remove some of the redundant summary of results, and add a paragraph to cover these instead. For example, there are a number of studies on Centrobin, Cep120 and Neurl4 that would warrant discussion here. Both Centrobin and Cep120 show daughter-centriole enrichment in mammals, and both show a similar loss from mother centrioles and enrichment on daughter centrioles coincident with procentriole assembly (e.g. Zou et al, JCB 2005; Mahjoub et al, JCB 2010). However, this occurs at the G1-S transition, which is NOT the stage at which centriole-to-centrosome conversion occurs. Thus, mechanisms for this translocation
must exist outside of mitosis, and may be regulated by other kinases. Moreover, altering the localization of proteins such as Cep120 have been shown to impact accumulation of PCM proteins and microtubule dynamics in mitosis (e.g. Comartin et al, Current Biology, 2013) and in interphase (e.g. Betleja et al, eLife 2018). Thus, I think it is important to discuss the current results in the broader context. I believe the asymmetric regulation of centriolar and centrosomal proteins is an important topic; thus highlighting these studies and how they relate to what is known in the field in general would be very beneficial.

We appreciate this insightful comment and added a short summary paragraph to the discussion. Since our manuscript predominantly focuses on Centrobin and its regulators, we focused the additional discussion on mammalian Centrobin.

Rev. 2:
In this elegant paper the authors show that establishment of Polo and Centrobin (Cnb) asymmetry between engaged mother (low levels) and daughter (high levels) centrioles at the apical centrosome during mitosis in Drosophila neuroblasts is important for the establishment of centrosome asymmetry in the following interphase, which is itself necessary for subsequent spindle alignment. This is an important area of research as it directly relates to how asymmetric stem cell divisions are regulated. The authors use cutting edge super-res imaging and very powerful genetic tools to provide beautiful observations and establish their model. These tools will be of great value to others in the centrosome filed. The authors also reveal some mechanistic insight by providing good evidence that the asymmetry is established at least in part by a direct transfer of molecules from the mother to the daughter centriole. From looking at the pre-print version of this paper, it is clear this version
of the paper has been improved by including the light-sensitive ectopic recruitment experiments that prove establishment of asymmetry during mitosis is necessary for proper asymmetry in the following interphase. The data is very clearly presented - I commend their Figure presentation, it is excellent and very helpful to the reader due to the complexity of the subject. I fully support publication in PloS Biology after the authors address my relatively minor concerns below.

1) "Cartwheels started to duplicate in late telophase, manifested in the appearance of a third Sas-6 positive cartwheel (blue arrowhead in Supplementary Fig.1c). Based on these data we conclude that in third instar larval neuroblasts centriolar cartwheels are duplicated in early interphase, forming a new procentriole. This procentriole subsequently converts into a mature centriole during mitosis through progressive loading of Asl. Thus, by the end of telophase, both neuroblast centrosomes contain two replication competent mature centrioles, an older mother and younger daughter centriole, which separate in the following interphase starting the cycle again." Did the authors always see that one centriole duplicated before the other in telophase (as seen in FigS1C)? If so, do they know which is destined to be the apical centrosome? I wonder in part because photoconversion results from Januschke et al., 2011 indicated that "at the time when apical and basal centrosomes split in
Drosophila NBs, the non-motile apical centrosome contains only one centriole."

This is an interesting point and we are interested in pursuing this in the future. In brief, in telophase, we always find one centriole duplicated before the other but cannot assign whether this occurs on the apical or basal centrosome. Such a ‘duplication asymmetry’ can also be seen in early interphase although more cells show both centrioles already duplicated. Thus, the data suggest that centriole duplication is asymmetric and starts in telophase. However, we don’t know (yet) whether this occurs on the apical or basal centrosome. This data is preliminary and is not suitable for the current manuscript.

Also, Januschke’s 2011 study used spinning disc microscopy, which cannot achieve the same resolution as the SIM microscope we used here. Thus, it is difficult to compare the results accurately.

2) Authors should point out that while Polo and Cnb seem to be co-dependent, it is still possible to have Polo without Cnb because this is true of the mother centriole in the basal centrosome.

We agree that Polo can be localized on the mother centriole of the basal centrosome without Cnb. However, we refer to a co-dependency of Polo and Cnb during the dynamic localization in mitosis, when Polo and Cnb ‘transfer’ from the mother to the daughter centriole. This pattern can be observed on both centrosomes during mitosis as both Cnb and Polo enrich on the daughter centriole. Loss of Cnb also affects Polo localization on both centrosomes.

3) "Loss of Wdr62 or Cnb also affects asymmetric centriolar Polo localization. Yet, interphase centrosomes lose their activity in these mutants. wdr62 mutants and cnb RNAi neuroblasts both show low Polo levels in interphase (Ramdas Nair et al., 2016). We thus hypothesize that in addition to an asymmetric distribution, Polo levels must remain at a certain level to maintain interphase MTOC activity; high symmetric Polo results in two active interphase MTOCs whereas low symmetric Polo results in the formation of two inactive centrosomes. Indeed, our optogenetic experiment triggered an increase in centriolar Polo levels upon blue light induction, suggesting that both Polo levels and distribution influence MTOC activity." I think the link between low Polo levels and low MTOC activity can easily be explained by the findings that phosphorylation of Cnn by Polo causes Cnn to oligomerise into a scaffold that supports PCM assembly (Conduit et al., 2014, Dev Cell). This is likely also
true at interphase apical centrosomes in neuroblasts. Indeed, it was shown that an asymmetry in Cnn occurs during early interphase and that Cnn is required for proper retention of the apical centrosome (Conduit and Raff 2010). The authors should discuss this.

We thank the reviewer for the comment. We agree with this statement and included it in the discussion.

4) "In early prophase neuroblasts, Polo was localized on the existing centriole on both centrosomes (Fig.4a,b & (Ramdas Nair et al., 2016))". Should this read "existing mother centriole"?

This is correct but to avoid confusion we decided to just use the phrase ‘existing centriole’.

5) "Centrosome asymmetry has previously been described to occur in asymmetrically dividing Drosophila neural stem cells (neuroblasts), manifested in biased interphase MTOC activity and asymmetric localization of the centrosomal proteins Cnb, Plp and Polo (Lerit and Rusan, 2013; Ramdas Nair et al., 2016; Singh et al., 2014; Januschke et al., 2011; 2013)." I think here the authors should also cite Conduit and Raff 2010 and include Cnn in the list of proteins where asymmetry has been observed.

We thank the reviewer for the comment and included this reference.

Rev. 3:
The study by Gallaud et al addresses the timing and mechanisms of establishment of an asymmetric identity between centrioles during the centrosomal cycle in drosophila, using the asymmetric division of larval neuroblasts, which are self-renewing stem cells in which the stereotyped orientation of the axis of division functionally relies on this asymmetry.

While the very mode of duplication of centrioles means that there is no ambiguity as to which is the mother and which is the daughter centriole, the question of how and when a daughter centriole acquires the molecular make up and identity of a mother is not entirely clear, nor is the dynamics of how this controls their differential behaviour. In the case of the dividing NB, this identity is manifest in the ability of one centriole to build an MTOC activity during the next cell cycle.

It was already known that larval NBs inherits the "youngest" centrosome from the previous cell cycle, and that on this centrosome, Centrobin (Cnb) localizes on the daughter centriole, which allows this centriole, after separation, to reconstitute an active MTOC activity, while its mother has no MTOC activity. It was also known that phosphorylation of Cnb by the polo kinase regulates its localization and activity. The current study addresses two questions:

- when does Cnb accumulate on the newly synthetized daughter centrioles, and disappear from the centriole that transits from a daughter to a mother identity.

- To which extent is the timing of this Cnb relocalization and accumulation at the time of mitosis important for the differential MTOC activity

Their main results/conclusions and advances are as follows:

1) A very detailed description of the dynamics of Cnb localization during the NB cell cycle. As the initially daughter/Cnb+/apical/MTOC+ centriole starts to duplicate upon mitotic entry, Cnb disappears from this centriole (that is transiting to a mother centriole) and almost simultaneously accumulates on the new daughter while it is being produced.

2) FRAP experiments suggest that at least part of Cbn on the new daughter derives from a relocalization of the pool that was initially present on its mother although an additional, newly synthetized pool of Cbn also participates in late mitosis. As the older mother/Cnb-/basal/MTOC- centriole duplicates to produce a new daughter, a new pool of Cnb accumulates on this new daughter. All these changes occur during mitosis

3) Phosphorylation of Cnb by polo contributes to (although apparently, at least in the genetic backgrounds used in the study, not 100% required for) its clearance from the "old" daughter, but is not required for its accumulation on the new daughters, which the authors summarize as "Polo dependent phosphorylation of Cnb is necessary for the establishment of molecularly distinct centrioles during mitosis, impacting subsequent molecular interphase asymmetry."

4) The localization of polo resembles that of Cnb, being cleared from the older apical centriole and accumulating on its daughter as it is formed during mitosis; it follows a similar dynamic on the basal centrosome. Its clearance from older and asymmetric accumulation on daughter centrioles reciprocally depends on Cnb;

5) Optogenetic ectopic recruitment of polo or Cbn to the mother centriole during mitosis is sufficient to prime an ectopic MTOC activity during the following interphase. However it is not necessarily required, as recruitment during interphase also results in high MTOC activity.

Although it is well written, the study is sometimes difficult to follow, as the distinctions between centriole and centrosome can be confusing, and it is not always immediately clear whether the terms mother and daughter refer to the previous or current cell cycle. But it is an inherent difficulty to this field, which requires very careful reading.

The functional approaches, based on gain of localization, are clever and beautifully performed. Hence one cannot help being slightly frustrated, as the authors may also feel, by some of the results that provide interesting hypotheses but are not entirely conclusive, such as those of the FRAP data or the optogenetic data.

Nonetheless, overall this is a beautifully detailed description of the dynamic change from daughter to mother centriole identity in the NB asymmetry context. The fine-grained temporal analyses, both in the purely descriptive data and for the functional approaches, provide a significant improvement over what had been shown in earlier studies on the same genes and proteins. Although the context is very specific, this study will in principle be of interest to a broad readership of cell biologists interested in the cell cycle, centrosome/centriole duplication, and more generally organelle reproduction; as well as to developmental/stem cell biologists with an interest in the molecular basis of centrosome asymmetry and its role in fate determination beyond the fly community.

Below are a few suggestions and some questions that should be addressed, and minor points

Concerning the set of optogenetics data: how stable is the ectopic recruitment of polo or Cbn to the mother centriole? The dissociation is only shown for a Cherry-tagged SspB (fig S5), but it is possible that although polo or Cbn are not normally present or recruited to the mother centrosome, an ectopic recruitment via the optogenetic approach would lead to a stable maintenance even after the stimulus is switched off. The interpretation of the "priming" of the strong MTOC activity would therefore be different, as in one case it would imply that the transient presence of polo or Cbn has stably induced a change in the mother centriole, whereas the other case would just mean that polo and/or cbn can directly induce the MTOC activity (as is suggested in Fig S5b, where the stronger recruitment of polo in prophase already induces a strong MTOC activity in the cell -judging on Jupiter-cherry signal), but not necessarily that they primed the centriole during mitosis for MTOC
activity later on. Therefore, it would be important to monitor and describe the localization of SspB-polo and SspB-Cnb after optogenetic stimulation is stopped.

This is a good suggestion but currently not technically possible. Polo is tagged with EGFP and Cnb with mDendra2, both of which are visualized with blue light. We cannot follow Polo or Cnb retention dynamics without also perturbing their normal localization dynamics.

Furthermore, the current model suggests that Cnb and Polo act in interphase to regulate biased MTOC activity. Perturbing the mitotic localization dynamics of Polo and Cnb in mitosis should not affect interphase MTOC activity. The idea of a stable change is interesting but considering all the data of the manuscript – especially the dynamic localization of Cnb and Polo as shown in Figure 1 and 4 – makes the priming model more plausible.

Along the same line, experiments in which Cnb or polo would be destroyed (eg using the AID degradation system or adaptation of the deGradFP system) specifically during mitosis or interphase would be more informative, although I do not know whether the temporal dynamics of such approaches is compatible with the short cycle time of the NBs

Unfortunately, the deGradFP system takes too long to remove a protein of interest. For instance, it takes ~ 1h to deplete 50% of a YFP-tagged target protein (see Caussinus et al., 2011; Figure 3b).

What happens to Cnb localization in PACt-vhhGFP4 + polo::EGFP? Is symmetric MTOC activity lost in PACt-vhhGFP4 + polo::EGFP in a Cnb-/- background?

Since we don’t have a mCherry::Cnb line, we cannot do this experiment. Given the Cnb localization depends on Polo phosphorylation, we assume that Cnb asymmetry in mitosis would be perturbed in this experimental condition.

Also, we have not perturbed Polo localization in a *cnb* mutant background, which is an interesting experiment. Given that Polo is still localized on centrosomes in *cnb* mutant neuroblasts but its asymmetric distribution is perturbed, we would speculate that Polo trapping would not be sufficient to restore interphase MTOC activity, unless Polo levels would increase.

Minor points:
In fig 6k, only the apical centrosome has strong levels of polo in ctrl cells and it is almost completely absent in the basal centrosome; However in figure 4b polo is present on the basal mother centriole in prophase and disappears in prometa. So is there a transient increase in prophase before it disappears?

Figure 6k refers to an interphase neuroblast whereas Figure 4 shows a mitotic neuroblast. Polo levels increase during mitosis on both centrosomes. So yes, there is an increase in Polo on both the apical and basal mother centriole. The data in Figure 4b suggests that Polo either relocalizes from the mother to the daughter centriole or disappears on the mother and becomes enriched on the daughter.

S1C: Asl seems to display very similar intensities on both centrioles in the Sas6-GFP flies, whereas it is more asymmetric in the other experiments (eg Cnb-YFP). Could there be an artifact from using Sas6-GFP?

Either this or a difference in the display mode. For Figure S1C, the emphasis was on centriole duplication. We used an interpolation mode to display the data, which somewhat smoothened the display of the markers. For the other figures, we removed the interpolation mode to display the data more accurately.

Fig3A-B: The quantified timeline displayed in 3B is a bit misleading. It is fixed material with no way to distinguish between apical/basal and old/young centrosome. So the dichotomy is established for each neuroblast and the centrosome whose mother centriole displays the highest amount of Cnb falls into the "strong delay category". As an obvious result, all centrosomes qualified as "high Cnb" ones will constitute a category displaying a stronger delay than the "low Cbn" one. This assumes an irreversible and coordinated progression (even if shifted in time) between the two centrosomes from mother with Cnb to mother without Cnb. This may be the case but should be explained.

Indeed, in this experiment, the apical/daughter and basal/mother centrosome stereotyped pattern is altered because of MTOC loss leading to centrosome inheritance randomization.

In the wild-type situation show in figure 1, the Cnb ratio is the strongest on the basal centrosome because it was absent on the mother centriole and get recruited on the daughter centriole only (all or nothing).

Therefore, in order to compare the phosphomutant with the control, we reasoned that the centrosome 2 should be the one with the strongest asymmetry ratio which is the weaker delay in achieving full asymmetry.

We believe this is the best way to compare with control to show that: (1) around 68% of neuroblast start mitosis with unphosphorylable Cnb on both centrosomes, and (2) there is a delay to complete the full establishment of asymmetry since by telophase, around 71% of neuroblast has at least one centrosome for which Cnb ratio is below 10 (only 3% in a control).

Fig3C-G: these results are difficult to correlate with the 3D-SIM data. 3B show us that about 2/3 of cases display a normal "apical" type of Cnb distribution (adding centrosomes 1 and 2) while 3G, although at a later time point , shows an almost inverse 3/4 in favor of double Cnb cases. Please explain

We see this differently for two reasons: (1) in telophase, Cnb seems to be present in both the ‘strong’ and ‘weak’ delay category, which does not occur in wild type (see Figure 1g). If we add up these bars, there are about 2/3 of cases with abnormal Cnb distribution. (2) Similarly, superresolution images of the same genetic background of interphase neuroblasts shows us ~ 2/3 of neuroblasts (data not shown, see below), which contain 2 Cnb positive centrioles. In wild type neuroblasts, there is always only 1 wild type centriole. Thus, the 75% of two Cnb+ centrioles shown in 3G is consistent with this result. This data shown below is not included in the manuscript.

Lines 75-76: very unclear phrasing…

We have rephrased this statement, hoping it will provide more clarity.

Lines 145-7: "Cartwheels started to duplicate in late telophase, manifested in the appearance of a third Sas-6 positive cartwheel (blue arrowhead in Supplementary Fig.1c). Based on these data we conclude that in third instar larval neuroblasts centriolar cartwheels are duplicated in early interphase, forming a new procentriole." These conclusions are a little strong. Why do we see only one additional Sas6 dot and not two? How often do the authors see a Sas6 second dot on both centrioles and when does this second dot appears? Could the author give us an approximate duration of the interphase?

We have observed late telophase cells with two, three, but never four Sas6 positive procentrioles. In early interphase, we find neuroblasts with three or four Sas6 positive procentrioles. These results suggest that centriole duplication is asymmetric and starts as early as late telophase.

In general, neuroblasts stay in interphase between 40-120 minutes. These are preliminary results and we are interested in pursuing these observations in the future.

Line 232: "Since we cannot accurately distinguish between apical and basal centrosomes in cnb mutants expressing YFP::CnbT4A,T9A,S82A" A very brief reminder of the Cbn LOF could be useful to the reader.

We thank the reviewer for the comment and modified the manuscript accordingly.

Movies: for most of them the relevant centrioles/centrosomes are difficult to follow, please use some arrows or outlines

We have modified all movies accordingly.


Fig7B: The diagram is clear at the individual level but it is difficult to understand the logic of the numerous different light activation regimes, with apparently very different illumination durations. A little clarification would be useful.

This is a valid comment and we have modified the figure legend accordingly
